# Supplementary material for: Profiling immuno-metabolic mediators of vitamin B12 deficiency among metformin-treated type 2 diabetic patients in Ghana
Source: PLoS One. 2021 Mar 30;16(3):e0249325. doi: 10.1371/journal.pone.0249325 (PMC8009370; doi:10.1371/journal.pone.0249325)
Supplement: S1 File — (DOCX) [file pone.0249325.s001.docx]

**S1 Table: Comparison of select hematological parameters based on** **vitamin B_12_ status**

| **Hematological parameters** | **Non-deficient** | **Deficient** | **p-value** |
| --- | --- | --- | --- |
| TWBC (10^3^/𝜇L) | 5.16±1.81 | 5.07±1.57 | 0.715 |
| RBC (10^6^/𝜇L) | 4.01±0.61 | 4.03±0.58 | 0.830 |
| Hemoglobin (g/dL) | 12.11±1.55 | 11.98±1.52 | 0.574 |
| HCT (%) | 33.97±5.43 | 34.35±3.67 | 0.593 |
| MCV (fL) | 82.87±7.30 | 84.15±8.80 | 0.278 |
| MCH (pg) | 30.75±2.51 | 30.92±2.35 | 0.646 |
| MCHC (g/dL) | 36.49±1.86 | 36.49±1.90 | 0.654 |
| PLT (10^3^/𝜇L) | 184.0 (150.0-210.0) | 192.0 (158.5-213.0) | 0.373 |

**S2 Table:** **Association between the duration of treatment with metformin, daily dose of metformin and diabetic neuropathy**

|  | **MNSI-Q** | |  |  |
| --- | --- | --- | --- | --- |
| **Variable** | Neuropathy absent  (n = 135) | Neuropathy present (n = 65) | cOR (95% CI) | *p*-value |
| **Metformin dosage**  **(mg/day)** |  |  |  |  |
| 1000-2000 | 127 (78.4) | 35 (21.6) | 1 |  |
| > 2000 | 8 (21.1) | 30 (78.9) | 13.61 (5.73-32.32) | **< 0.0001** |
| **Duration of treatment**  **with metformin (years)** | |  |  |  |
| < 5 | 13 (72.2) | 5 (27.8) | 1 |  |
| 5-10 | 44 (73.3) | 16 (26.7) | 0.95 (0.29-3.08) | 0.9260 |
| > 10 | 78 (63.9) | 44 (36.1) | 1.47 (0.49-4.39) | 0.4930 |
|  | **MNSI-PE** | |  |  |
| **Metformin dosage**  **(mg/day)** |  |  |  |  |
| 1000-2000 | 159 (98.1) | 3 (1.9) | 1 |  |
| > 2000 | 28 (73.7) | 10 (26.3) | 18.93 (4.90-73.11) | **< 0.0001** |
| **Duration of treatment**  **with metformin (years)** | |  |  |  |
| < 5 | 18 (100.0) | 0 (0.0) | 1 |  |
| 5-10 | 58 (96.7) | 2 (3.3) | - | 0.9980 |
| > 10 | 111 (91.0) | 11 (9.0) | - | 0.9980 |

cOR: crude Odd Ratio; MNSI-Q: Michigan Neuropathy Screening Instrument Questionnaire; MNSI-PE: Michigan Neuropathy Screening Instrument Physical Examination. *p*-values calculated by univariate logistic regression

**S3 Table: Association between the duration of treatment with metformin, daily dose of metformin and vitamin B12 deficiency**

|  | **Vitamin B12 deficiency status** | |  |  |
| --- | --- | --- | --- | --- |
| **Variable** | **Non-deficient (n=119)** | **Deficient (n=81)** | **cOR(95% CI)** | ***p*-value** |
| **Metformin dosage**  **(mg/day)** | |  |  |  |
| 1000 | 20 (62.5) | 12 (37.5) | 1 |  |
| 2000 | 85 (65.4) | 45 (34.6) | 0.88 (0.40-1.97) | 0.760 |
| 3000 | 14 (36.8) | 24 (63.2) | 2.86 (1.08-7.56) | **0.034** |
| **Duration of treatment**  **with metformin (years)** | |  |  |  |
| < 5 | 12 (66.7) | 6 (33.3) | 1 |  |
| 5-10 | 34 (56.7) | 26 (43.3) | 1.53 (0.51-4.62) | 0.451 |
| > 10 | 73 (59.8) | 49 (40.2) | 1.34 (0.47-3.82) | 0.581 |

cOR: crude odd ratio, *p*-values calculated by univariate logistic regression

**S4 Table: Vitamin B12 deficiency using serum B12 levels**

| **Serum B12 levels (pmol/l)** | **Total number** | **Percentage** |
| --- | --- | --- |
| <148 | 108 | 54.0 |
| ≥148 | 92 | 46.0 |
