# Supplementary material for: Profiling immuno-metabolic mediators of vitamin B12 deficiency among metformin-treated type 2 diabetic patients in Ghana
Source: PLoS One. 2021 Mar 30;16(3):e0249325. doi: 10.1371/journal.pone.0249325 (PMC8009370; doi:10.1371/journal.pone.0249325)
Supplement: Attachment — Submitted filename: Response to Reviewers comment.docx [file pone.0249325.s002.docx]

The Editor,

PLOS ONE

Dear Sir/Madam,

**RESPONSE TO REVIEW COMMENTS**

The authors very much appreciate the timely and scrupulous review of our manuscript (PONE-D-20-39903). Kindly find below the response to the reviewers’ comments. Tracked changes have been employed to highlight texts revised per the reviewers’ recommendations. Revised statements indicated in this response are in “quotation marks”.

**Reviewer #1**

**Comment 1:** It is a relatively small study (n=200) and there is no control group.

**Response:** Thank you for your comment. This observational study is a baseline study to evaluate to use of Fedosov combined marker in Ghanaian population which has not been explored. However, a case-control study with large sample size will add a layer of knowledge. We acknowledged this as a limitation worth exploring by future studies (line 433-4).

“Another limitation of this study is the relatively low sample size and the cross-sectional nature of the design. Further case-control and prospective studies are warranted.”

**Comment 2**: There is a very high prevalence of Vitamin B12 deficiency (40.5%) reported, but in the absence of any control (for example, age/gender matched individuals not on Metformin) it is difficult to attribute this high prevalence only to metformin use. There are numerous other prior studies that are larger, with appropriate controls, which show lower prevalence.

**Response:** Thank you for your comment. Although this observational study reported a very high prevalence of vitamin B12 deficiency (40.5%) with the absence of a control group compared to other studies conducted elsewhere, this was not unexpected for reasons such as disparities in environmental and genetic factors that may impact their levels. Additionally, differences in the parameters (MMA, serum B12, etc) considered in determining B12 deficiency is another factor. In an effort to accurately capture patients with true vitamin b12 deficiency, we relied on the combined 4cB12.

**Comment 3**: In the absence of a control group, a before treatment B12 compared to B12 after treatment with Metformin would provide useful data. This was not done as this is an observational study.

**Response:** Thank you very much for your comment. The current study was a cross -sectional study where one-point sample was taken. We have highlighted this as a limitation (line 433-4).

**Comment 4**: The study uses a relatively little used and poorly validated computational model (Fedosov combined marker) to diagnose Vitamin B12 deficiency. Low B12 levels alone are a sensitive indicator of deficiency (up to 95% sensitive depending on cutoff) (NEJM, 2013. 368:149-60). In those with borderline low Vitamin B12 (200-300 pg/ml), an elevation of MMA and Homocysteine add to the sensitivity, but both need to be elevated as homocysteine alone can be high in folate deficiency. MMA elevations may not be specific as other conditions including renal disease can cause elevations. Therefore, these should be used in situations where Vitamin B12 is borderline low. Holotranscobalmin is neither specific nor sensitive but can be useful in pregnancy. There is little data on validity of Fedosov model (Reference 18 in current article) which has been cited only 15 times since its publication in 2013. Reading the original paper reveals this is a theoretical construct which is validated against cognitive impairment in the elderly and hematological impairments.

**Response:** Thank you for your comment. Although serum B12 presents a good marker for B12 deficiency, several studies have highlighted inconsistencies in using serum B12 to define B12 deficiency. High serum B12 levels can be accompanied by signs of deficiency, and functional deficiency from tissue uptake defects and action of vitamin B12 at the cellular level have been implicated in this association [PMID: 26807790; PMID: 21733877]. There is also evidence that functional vitamin B12 deficiency can occur regardless elevated serum B12 levels and using MMA alone is limited. [PMID: 21733877]. Moreover, elevation of MMA is non-specific in the presence of renal impairment (a somehow common sequela in diabetes) but the use of MMA and homocysteine enhance the sensitivity to identify individuals with borderline vitamin B12 deficiency. Thus, the most accurate marker to true B12 deficiency remains to be identified. However, we believe that a model such as Fedosov 4cB12 which accommodates other markers in addition to serum B12 provides an opportunity.

**Comment 5**: The data correlating B12 deficiency with neurological impairments (two clinical measures of peripheral neuropathy) and immunological parameters does not provide any new information.

**Response:** Thank you for your comment. Studies correlating vitamin B12 status to diabetic neuropathy has produced conflicting results [PMID: 27730072; PMID: 28882470; PMID: 27716423]. The two clinical measure of peripheral neuropathy in this study will contribute to the understanding of the relationship between vitamin B12 deficiency is associated and risk of neuropathy.

**Comment 6**: This study shows rates of Vitamin B12 deficiency (40.5%) which is far higher than almost all prior studies, but the non-standard measure (Fedosov wellness quotient) may be the reason for this discrepant finding.

**Response:** Thank you very much for your comment. The high prevalence of vitamin B12 deficiency reported in our study (40.5%) is not extremely different from a related study in Ghana which reported high prevalence of B12 deficiency (32.1%) among T2DM patients on metformin [DOI: 10.1080/20905068.2019.1662647]. Additionally, similar to vitamin D deficiency which is very common in the study region despite adequate sunshine, our pilot study found that the use of only serum B12, which is commonly used in other populations overestimates B12 deficiency as evidenced in the higher prevalence of B12 deficiency (>50% of the population) based on serum B12 (Supplementary file: Table S4). In an effort to normalize and accommodate all relevant markers, we used the Fedosov wellness quotient.

**Comment 7**: Since the average B12 in the group, on page 14, line 287 is reported as 142 pmol/L (which is below the threshold for severe Vit B12 deficiency 148 pmol/L or 200 pg/ml), it would be informative to know what was the percentage of B12 deficiency using just the threshold of 148 pmol/L which is very specific cutoff.

**Response:** Thank you for your comment. As indicated in the response to comment 6, the use of serum B12 only in the study setting overestimates the prevalence of B12 deficiency, corroborating previous reports on the disparity between serum B12 levels and functional B12 deficiency (Kindly refer to response to comment 6 and Supplementary file: Table S4).

**Comment 8**: The study needs to emphasize what new knowledge it adds to the topic of B12 deficiency in Metformin treated patients.

**Response:** Thank you for your comment. Specifically regarding B12 deficiency in diabetes, as highlighted in line 120-3, the use of individual markers (serum B12, MMA, hTC, Hcy) has rather yielded inadequate reflection of true vitamin B12 status. The study thus highlights the likelihood of missing some cases of B12 deficiency when a single indicator is used compared to using multiple markers (line 120-3 & 378-80).

“Of note, the high rate of vitamin B12 deficiency in this study compared to previous studies could be suggestive that the use of individual markers (serum B12, MMA, hTC or Hcy) underestimates the vitamin B12 status among diabetic populations.”

**Minor comment:**

**Comment 1:** Labelling/legends in figure 1 need to be clearer

**Response:** Thank you for the observation. Labelling/legend in figure 1 has been made clearer.

**----------------------------------------------------------------------------------**

**Reviewer #2**

**Major comments:**

**Comment 1**: It would be appropriate to clarify how the diagnosis of type 2 diabetes was made. The presence of gastric parietal cell antibody makes it imperative to exclude type 1 diabetes

**Response:** Thank you for your observation. The inclusion and exclusion criteria have been clarified (line 177-86)

“The diagnosis of type 2 diabetes mellitus was made based on American Diabetes Association (ADA) criteria (based on defective progressive insulin secretory on the background of insulin resistance using fasting plasma glucose ≥7.0 mmol/L; glycated hemoglobin ≥6.5%; oral glucose tolerance test 11.1 mmol/L)”

“Patients with type 1 diabetes (diagnosed based on high plasma glucose in addition to autoimmune indicators such as autoantibodies to insulin, autoantibodies to glutamate decarboxylase (GAD65)) were excluded.”

**Comment 2**: A comparator group such as patients without diabetes or patients with type 2 diabetes but who were not treated with metformin would be required to justify the conclusion that the prevalence of Vitamin B12 deficiency and peripheral neuropathy are high in patients treated with metformin in Ghana. Could there be the possibility that B12 deficiency may be high in the general population.

**Response:** Thank you for your comment. It is possible that there is generally high prevalence of vitamin B12 deficiency in the study population given that a previous study reported over 30% prevalence using serum B12. Additionally, using only serum B12 levels, over 50% of our study population was deficient. Given that this is a baseline study, we have acknowledged the limitation of the cross-sectional design and suggested further studies in this regard (line 433-4).

“Another limitation of this study is the relatively low sample size and the cross-sectional nature of the design. Further case-control and prospective studies are warranted.”

**Comment 3**: Is there any correlation between the dose/duration of treatment with metformin and Vitamin B12 deficiency/peripheral neuropathy?

**Response:** Thank you for your comment. Higher dose of metformin (3000mg/day) was associated with increased odds of vitamin B12 deficiency and neuropathy. However, the duration of treatment with metformin was not associated with vitamin B12 deficiency or neuropathy (Supplementary file: Table S2 & S3).

**Comment 4**: 2017 ADA guidelines recommended periodic monitoring of Vitamin B12 in patients treated with metformin over a long period of time, suggesting routine measurement in all patients treated with metformin may not be cost effective.

**Response:** Thank you very much for your comment. The sentence has been rephrased.

**Minor comment:**

**Comment 1**: Type II diabetes should be read Type 2 diabetes.

**Response:** Thank you for the comment. ‘Type II diabetes’ has been replaced with ‘Type 2 diabetes’.

**Comment 2**: “on metformin” is better rendered as “treated with metformin” throughout the manuscript.

**Response:** Thank you for the suggestion. “on metformin” has been replaced with “treated with metformin” throughout the manuscript.

**Comment 3**: Figure 4 appears confusing; a table might be easier to understand.

**Response:** Thank you very much for your comment. Due to the large number of biochemical and immunological parameters together with the five indicators of vitamin B12, using a table will make it very clumsy. However, further description has been added to make the figure clearer.

Thank you once again for the timely review and scrupulous of our manuscript. Looking forward to hear favorably from you.

Sincerely,

Samuel Asamoah Sakyi

**Corresponding author**
