# Supplementary material for: Profiling immuno-metabolic mediators of vitamin B12 deficiency among metformin-treated type 2 diabetic patients in Ghana
Source: PLoS One. 2021 Mar 30;16(3):e0249325. doi: 10.1371/journal.pone.0249325 (PMC8009370; doi:10.1371/journal.pone.0249325)
Supplement: Attachment — Submitted filename: Response to Reviewers comment vR2.docx [file pone.0249325.s003.docx]

The Editor,

PLOS ONE

Dear Sir/Madam,

**RESPONSE TO REVIEW COMMENTS R2**

The authors very much appreciate review of our manuscript (PONE-D-20-39903). Kindly find below the response to the reviewers’ and editor’s comments. Revised statements indicated in this response are in “highlighted in red”.

Editor’s comment

Please provide a detailed discussion of the limitation of your study with respect to the lack of a comparator group. This is critical. Your current two-sentence response is not sufficient: 'Another limitation of this study is the relatively low sample size and the cross-sectional nature of the design. Further case-control and prospective studies are warranted.'

You have to discuss the concerns about his lack of comparator group, and what your group should have done in terms of including a control group of healthy subjects, or a group of patients with diabetes but were not receiving metformin.

**Response:** The limitations has been discussed further to read “Another limitation of this study is the relatively small sample size and the cross-sectional nature of the design. The current study design lacked comparator and a control group of healthy subjects, or diabetes who are not on metformin, this makes it difficult to make causal inference. They are however, useful for establishing preliminary evidence in planning a future advanced study, subsequently, further case-control and prospective studies are warranted”. Page 20, line 430-435.

**Reviewer #2**

Reviewer #2: "It is imperative Ghana include periodic measurement of Vitamin B12 deficiency using the more sensitive combined indicators (4cB 12 ), in the management of T2DM patients treated with metformin" in concluding sentence of the abstract should read "if the findings of this study are verified in a prospective case-control study, it may be beneficial to include periodic measurement of Vitamin B12 using the more sensitive combined indicators (4cB 12 ) in the management of patients with T2DM treated with metformin in Ghana.

**Response:** The concluding sentence of the abstract and main manuscript has been revised to read " Upon verification of these findings in a prospective case-control study, it may be beneficial to include periodic measurement of Vitamin B12 using the more sensitive combined indicators (4cB 12) in the management of patients with T2DM treated with metformin in Ghana. Page 3, line 78-81

".

Thank you once again for the timely review of our manuscript. Looking forward to hear favorably from you.

Sincerely,

Samuel Asamoah Sakyi

**Corresponding author**
